# Therapeutic Efficacy of Lactonic Sophorolipids: Nanoceria-Assisted Combination Therapy of NSCLC using HDAC and Hsp90 Inhibitors

**DOI:** 10.7150/ntno.57675

**Published:** 2021-04-16

**Authors:** Shuguftha Naz, Tuhina Banerjee, Filbert Totsingan, Kalee Woody, Richard A. Gross, Santimukul Santra

**Affiliations:** 1 Department of Chemistry, Pittsburg State University, Pittsburg, Kansas 66762, United States.; 2 Department of Chemistry and Chemical Biology, Rensselaer Polytechnic Institute, Troy, New York 12180, United States.

**Keywords:** lactonic sophorolipid, HDAC inhibition, ganetespib, antioxidant nanoceria, cancer therapy

## Abstract

**Purpose:** Non-Small-Cell Lung Cancer (NSCLC) has gained resistance to common chemo- and radiotherapy due to the oncogenic K-RAS mutations. In this work, lactonic sophorolipids (LSL), a constituent of natural sophorolipids known to inhibit histone deacetylase (HDAC) activity, is used to evaluate its potential anticancer property for the treatment of NSCLC. In addition, ganetespib (GT), a Hsp90 inhibitor, is used for its known antitumor activity in several K-RAS mutant NSCLC cells. We propose, a functional anti-oxidant nanomedicine composed of nanoceria (NC) encapsulated with two-drug cocktail LSL and GT for the assessment of therapeutic efficacy of LSL and targeted combination therapy of NSCLC. NC is an excellent redox platform specifically used to supplement the therapeutic potency of these drugs to target both HDAC inhibition and Hsp90 signaling pathways in NSCLC.

**Methods:** Polyacrylic acid-coated nanoceria (PNC) was formulated and folic acid was conjugated on the surface of PNC using “click” chemistry to target NSCLC and to minimize adverse side effects. Solvent diffusion method was used for the encapsulation of individual drugs and co-encapsulation of drug-cocktail along with an optical dye DiI for diagnosis. We hypothesized that the therapeutic efficacy of LSL will be synergistically accelerated by the inhibition of Hsp90 mechanism of GT and redox activity of NC.

**Results:** For the targeted therapy of NSCLC, A549 cells were used and Chinese hamster ovary (CHO) cells were used as healthy control cells. Results showed more than 40% cells were dead within 24 h when treated with LSL nanodrug. When combined with GT, enhanced ROS signals were detected and more than 80% reduction in cell viability was recorded within 24 h of incubation. Treatments with NC without any drug showed minimal toxicity. Migration assays indicate that the highly metastatic nature of NSCLC is successfully restricted by this combination approach. To validate the effectiveness of this combination therapy various cell-based assays including detection of apoptosis, necrosis and HDAC inhibition of LSL were performed.

**Conclusion:** Functional nanoceria with drug-cocktail LSL and GT is successfully developed for the targeted treatment of undruggable NSCLC. The fluorescence modality helps monitoring the drugs delivery. Results demonstrate the potential therapeutic efficacy of LSL, which is synergistically accelerated by the Hsp90 inhibition mechanism of GT and redox activity of NC.

## Introduction

Histone acetylation and deacetylation plays a crucial role in the regulation of gene expression. These processes are balanced by histone acetylases (HAT) and histone deacetylases (HDAC) 1-3. Altered expression and mutation of genes associated with HDAC are directly linked to cell proliferation and tumor development 4. Studies have shown that HDAC inhibitors are known to alter gene expression, induce cancer cell apoptosis and death. Thus, the development of HDAC inhibitors, their therapeutic role and mechanism of action has received tremendous attention in the field of cancer treatment 5. Lactonic sophorolipid (LSL) is a glycolipid molecule synthesized from specific yeast species. Many reports are available on the immunomodulatory and anti-inflammatory properties of LSL 6-11. Furthermore, studies have reported the anticancer activity of LSL on different cell lines including pancreatic, esophageal and lung cancer. It is indicated that one of the mechanisms for anti-tumor activity of LSL is the HDAC inhibition, where histone deacetylases are inhibited interfering with gene expression 12-16.

Lung carcinomas are currently the leading cause of cancer-related mortality worldwide due to its' poor prognosis, multidrug resistance (MDR) and low survival rate 17-19. Over 85% of lung cancer cases are related to non-small-cell lung cancer (NSCLC) and the major histopathological subtypes are adenocarcinomas, squamous cell carcinomas and large cell carcinomas 18,20-24. Oncogenic K-RAS mutations in human NSCLC (10-30%) have gained resistance to chemotherapy and radiation 25-27. Although K-RAS mutations are well known, there is a lack of effective therapeutic options available for K-RAS driven NSCLC.^25^ Severe side effects, multidrug resistance and poor survival outcomes are the limitations of current NSCLC therapies 28,29, indicating the urgency of developing an effective therapeutic for its treatment.

Targeted delivery of two or more therapeutic drugs to tumor sites is showing promise for the effective treatment of cancer 30. Such combination therapies enable inhibition of multiple tumorigenesis pathways while overcoming severe side effects and multidrug resistance. Considering the importance of combination therapy, a new drug combination, LSL and ganetespib (GT) is proposed in this study. We reasoned that GT is a second generation Hsp90 inhibitor, which leads to the degradation of major client proteins, disrupts important signaling cascades responsible for cell proliferation and survival of tumor 31-34. GT is also believed to have antineoplastic activity and has shown promise for the treatment of NSCLC 35-37. In addition, the use of nanotechnology-based drug delivery system is an emerging area of interest for increased payloads, biocompatibility, and targeted delivery of chemotherapeutic drugs, resulting in minimal side effects. Recent studies indicate the huge potential of various nanoparticles as drug carriers in cancer therapy, including targeted drug delivery, diagnosis, imaging, and treatment 38-43. Among others, cerium oxide nanoparticles (NC) has received considerable attention due to its excellent catalytic and antioxidant properties with different cell systems. As an antioxidant, many reports are available indicating its cytotoxicity towards cancer cells while providing protective effects in certain neurodegenerative disorders 44-49.

**Figure 1:**
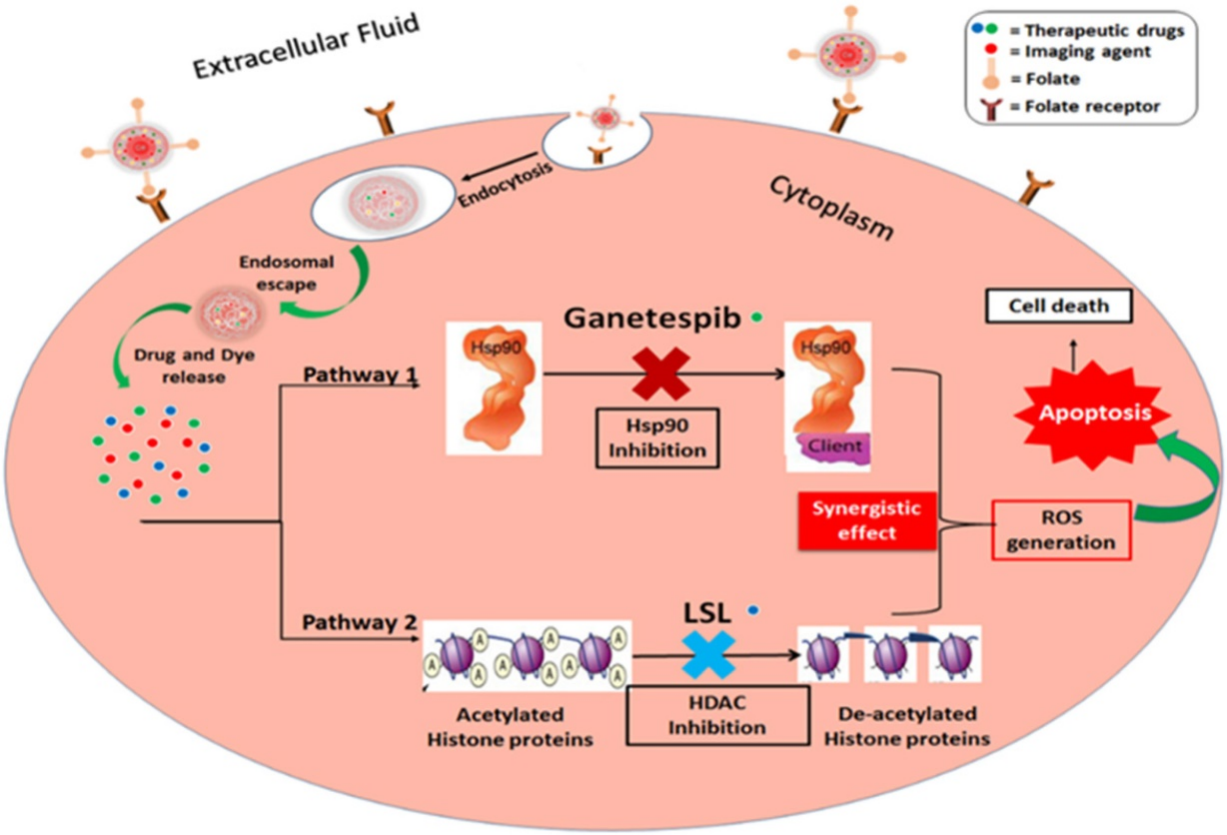
Schematic representation of the proposed mechanisms of action of the combination of ganetespib and lactonic sophorolipids for NSCLC treatment.

Herein, we report a new combination therapy approach for the effective treatment of NSCLC (A549 cells). The blend of chemotherapeutic drugs, LSL and GT, are encapsulated within folate conjugated nanoceria (FNC) to minimize their off-target effects. Additionally, the anti-oxidative nature of nanoceria facilitates for the survival of healthy tissues, while generating *in situ* chronic oxidative stress within tumor cells reducing their viability 50,51. This property of NC combined with the synergistic therapeutic actions where LSL and GT functions to inhibit HDAC and Hsp90 signaling pathways was evaluated as a treatment of NSCLC (**Figure 1**) 52,53. The therapeutic effects of this combination therapy were examined by performing various cell-based assays including cell viability, enhanced ROS, apoptosis and necrosis, migration and HDAC assays. Together, findings from this study provide clinically acceptable strong support for the developed NSCLC treatment that supports its further investigation as a candidate therapeutic option.

## Results and Discussion

### Synthesis and Characterizations of Functional Cerium Oxide Nanoparticles

**Figure 2:**
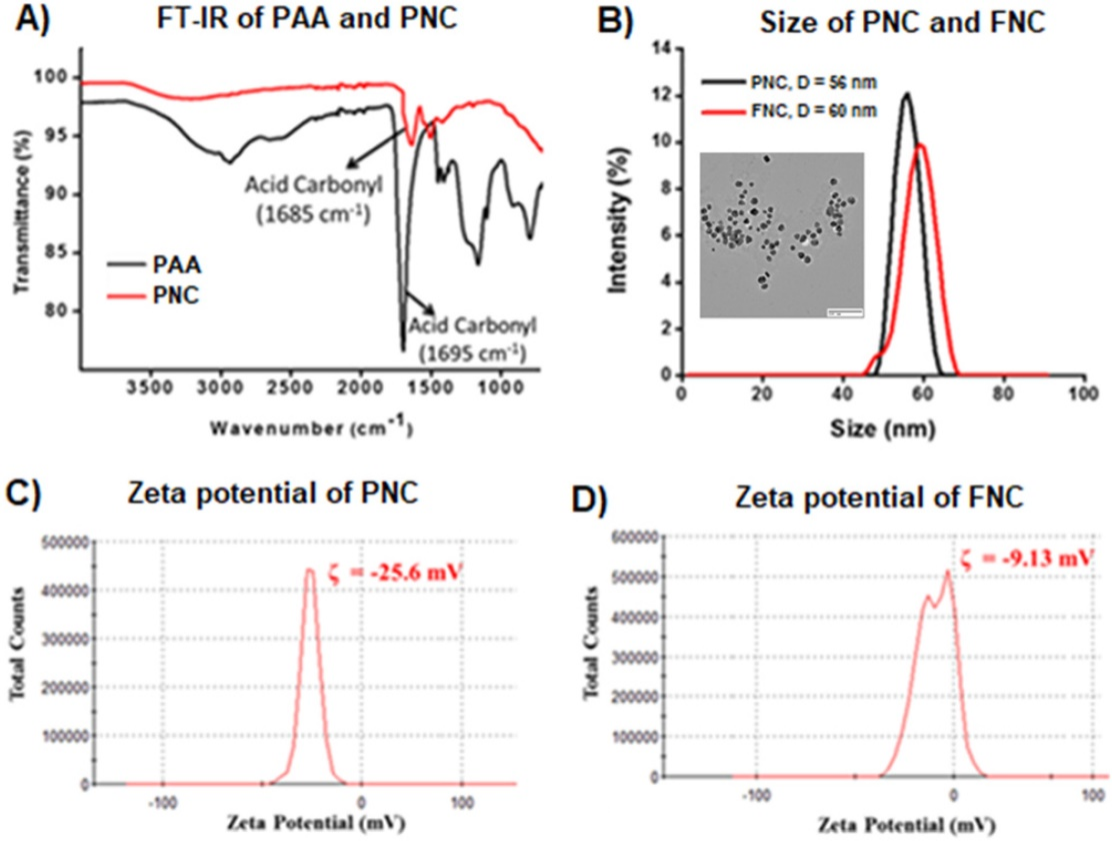
** A**) The presence of PAA coating of PNC particles was confirmed by the presence of an acid carbonyl band at 1685 cm^-1^ in the FT-IR spectrum. **B**) Dynamic light scattering (DLS) determined average diameter of PNC (56 nm) and FNC (60 nm), Inset: TEM image of PNC (scale bar: 100 nm) and zeta potentials of **C**) PNC and **D**) FNC.

**Scheme 1:**
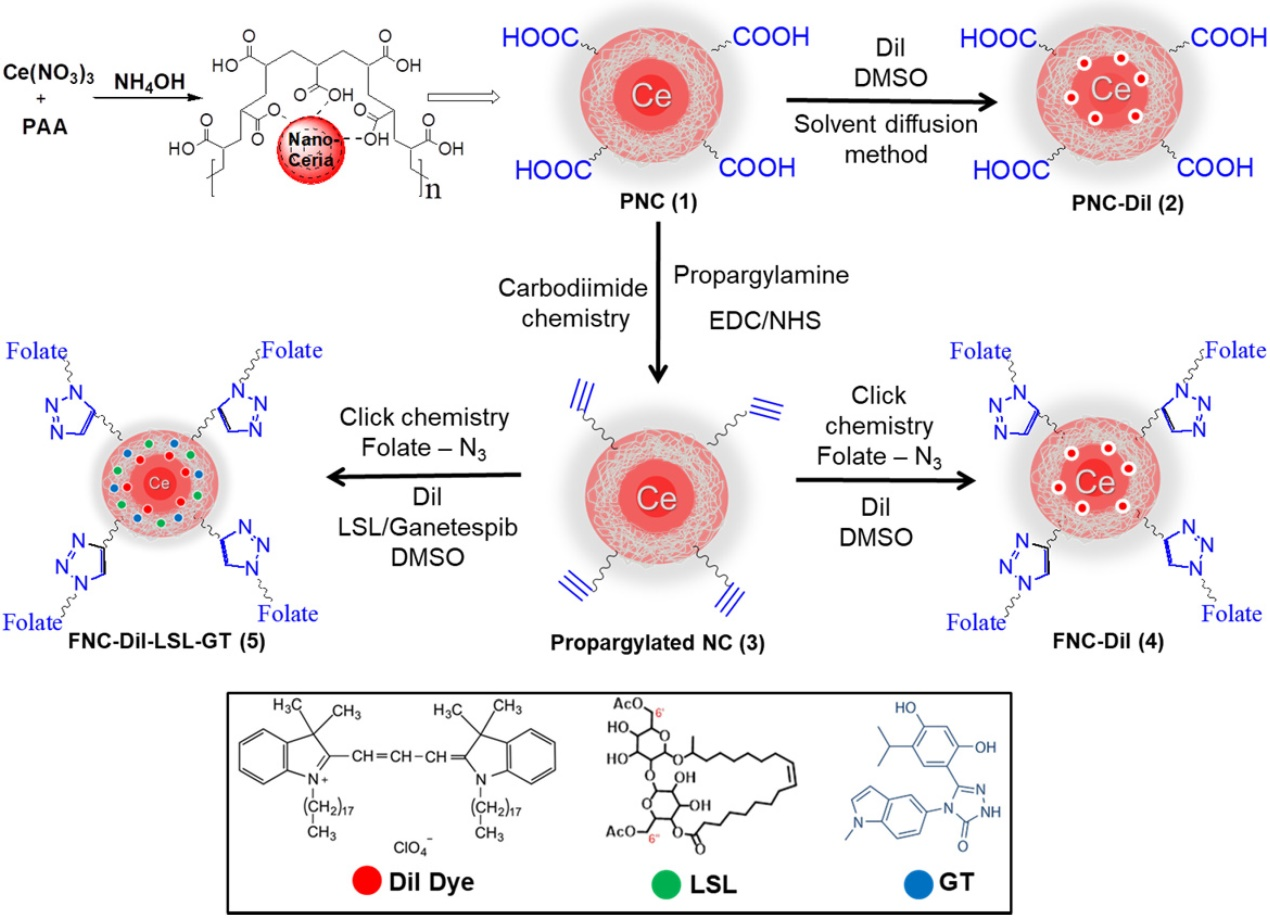
Schematic representation of the synthesis of functional NC. Polymer-coated nanoceria (PNC **1**) was synthesized using an alkaline precipitation method. Propargylated NC (**3**) was synthesized using carbodiimide chemistry and the “click” chemistry was used to functionalize **3** with folic acid (FNC, **4** and** 5**). Solvent diffusion method was used for the encapsulation of theranostics.

Carboxylic acid group functionalized cerium oxide nanoparticles (PNC **1**, **Scheme 1**) were synthesized using a previously reported water-based alkaline precipitation method 54. In summary, cerium nitrate salt was used as the source of nanoceria, whereas polyacrylic acid (PAA) was used as the stabilizing agent. The synthesized yellow PNC solution was then centrifuged to discard larger sized PNC particles. The resulting supernatant was dialyzed using a dialysis membrane (MWCO 6-8 kDa) to remove unreacted reagents including free PAA and NH_4_OH from PNC. The effective coating of NC by PAA was confirmed by vacuum drying and performing FT-IR analysis. As shown in **Figure 2A** (red line), the presence of a band at 1685 cm^-1^ confirmed the PAA coatings on PNC. The PAA coating results in a stable, water dispersible nanoparticle for efficient drug encapsulation. Furthermore, the surface carboxylic acid groups allow conjugation of various receptor-targeting molecules for targeted delivery. The average size (diameter) and overall surface charge (zeta potential) of PNC, determined by dynamic light scattering method, were D = 56 nm (black line, **Figure 2B**) and ζ = -25 mV (**Figure 2C**), respectively. The transmission electron microscopic (TEM) analysis showed the formation of highly dispersed cerium oxide core of 22±3 nm (Inset, **Figure 2B**).

**Figure 3:**
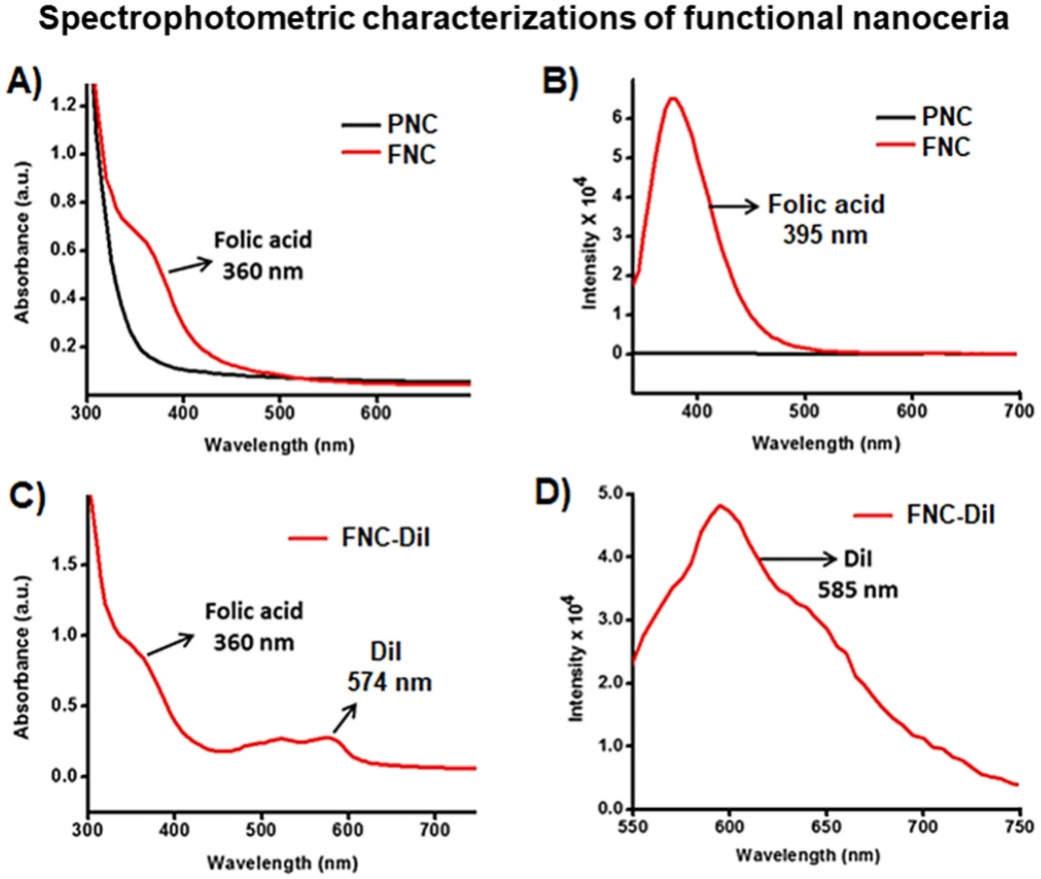
Characterization of functionalized NC. **A)** UV-Vis absorption and **B**) fluorescence emission spectra recorded from PNC (back line) and FNC (red line), indicated for the effectiveness of “click” chemistry. **C**) UV-Vis absorption spectrum of folate nanoceria and **D**) fluorescence emission at λ_em_ = 585 nm, confirmed for the successful encapsulation of DiI dye in FNC.

Conjugation of folic acid on the surface of nanoceria was carried out using “click” chemistry. First, the propargylated NC (**3**) and azide-functionalized folic acid were synthesized following previously published methods 14,15. Next, in the presence of a CuI catalyst, the conjugation of propargylated NC (**3**, 2.0×10^-3^ mol) and azide-functionalized folic acid (2.0×10^-2^ mol) was performed in phosphate buffered saline (PBS) solution at pH 8.0.^14,15^ The synthesized FNC (**4** and** 5,** 1.5×10^-3^ mol**, Scheme 1**) was purified by dialysis (MWCO 6-8 kDa). The successful folate conjugation was confirmed by performing FT-IR experiments and compared with FT-IR spectra of PNC and PAA polymer. As seen in **SI Figure S1**, compared to PNC, the FT-IR spectrum of FNC showed bands at 1690, 1605, 1490, 1411 and 3054 cm^-1^, which confirmed for the presence of folic acid in FNC. This was further confirmed by performing UV-Vis spectroscopy (**Figure 3**). Next, the fluorescent lipophilic cationic indocarbocyanine dye DiI (1.0 µM) was selected for optical imaging due to its higher extinction coefficient (ε > 125000 cm^-1^ M^-1^), red fluorescence emission (λ_max_ = 585 nm) and excellent photostability 55. This red dye was encapsulated by solvent diffusion method to formulate DiI-encapsulated FNC (**4**, 1.0×10^-3^ mol) which provided a positive control for optical imaging and treatment monitoring of cancer 15. The DiI dye loading was effective and the encapsulation efficiency (EE_DiI_) was 87%. Individual drug encapsulations (EE_LSL_ = 77%, EE_GT_ = 81%) and co-encapsulation of the therapeutic drug combination of LSL and GT (EE_LSL+GT_ = 75%) in folate nanoceria (**5,** 1.0×10^-3^ mol) was performed using similar modified solvent diffusion method. As described, in the solvent diffusion method of encapsulation, dye and drugs are encapsulated in the polymer (PAA) coatings of the nanoceria, instead of chemical conjugation. Hydrophobic-hydrophobic and intermolecular forces are the major driving forces for this encapsulation. Finally, these preparations were dialyzed for purification and stored at 4 °C for further characterizations. The overall diameter and surface charge of functional FNC (**5**), determined by DLS, are 60 nm and -9.13 mV, respectively (**Figure 2B** and** 2D**). The successful encapsulation of DiI dye in the polymer coating of FNC was confirmed by UV-Vis absorbance and fluorescence spectroscopy. Absorbance spectra of dye encapsulated FNC (**Figure 3A** and** 3C**) confirmed the presence of both folic acid (λ_abs_ = 360 nm) and DiI dye (λ_abs_ = 574 nm). This was further confirmed by their corresponding fluorescence spectra (Fol: λ_em_ = 395 nm; DiI: λ_em_ = 585 nm, **Figure 3B** and** 3D**). The overall size and fluorescence emission of these drug-loaded NC (**5**) preparations were found to be comparable over time (over 12 months), indicative of aqueous stability of functional NC in serum and PBS at physiological pH.

### Cargo release experiments

**Figure 4:**
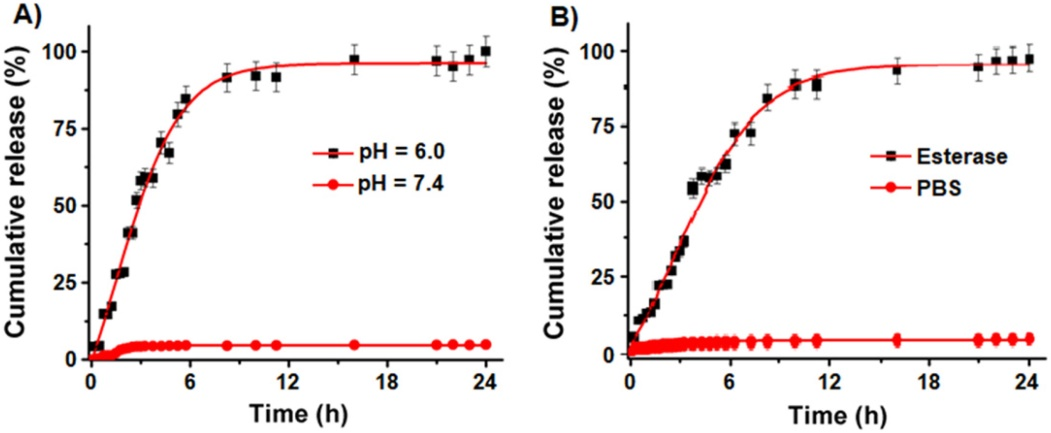
Controlled cargo release profiles of FNC-DiI-LSL-GT using dynamic dialysis method. **(A)** Controlled release of DiI dye from FNC with time in acidic pH (pH 6.0) was observed and about 80% of drug is released within 6 h of time. However, minimal release was seen at normal pH (pH 7.4). **(B)** Similarly, release of DiI dye in esterase enzyme (1.0 mM) was studied using fluorescence spectrometer (λ_em_ = 585 nm) where fluorescence maxima increased as the dye releases with time. However, there is no or minimal release of dye observed (red lines) at normal pH conditions.

To evaluate drugs and dye release kinetics of NC-based delivery system under external conditions representing the microenvironment of a tumor, we performed DiI dye release experiments under esterase enzyme and acidic pH conditions (pH 6.0). We have selected DiI dye as a representative cargo for this release study due to its strong fluorescence emission at 585 nm, whereas both the drugs LSL and GT are weak chromophores. As seen previously 31, the PAA polymer coatings of NC are disrupted in acidic environment by hydrolyzing the coordinate bonding between PAA polymer carbonyl groups and cerium oxide, thereby releasing the cargos. It is also important to evaluate the biodegradability of NC by incubating in the presence of esterase enzyme (1.0 mM) from porcine liver. Time dependent study of released cargos was determined using a dynamic dialysis method and measuring fluorescence emission at 585 nm using a microplate reader. The cumulative release of DiI dye at pH 6.0 (**Figure 4A**) indicated for a burst release of dye (80%) within 6 h of incubation and then the release was stabilized with time. The dye release was comparably slower when incubated in esterase enzyme (1.0 mM) as seen in **Figure 4B**. In contrast, at pH 7.4 no detectable release of dye was observed, which indicated for the stability of our cargos-loaded NC in physiological conditions. These results confirm that our NC drug delivery system releases the cargos within the acidic environment of tumor cells and are stable at normal pH.

### Cell viability assay (MTT assay)

**Figure 5:**
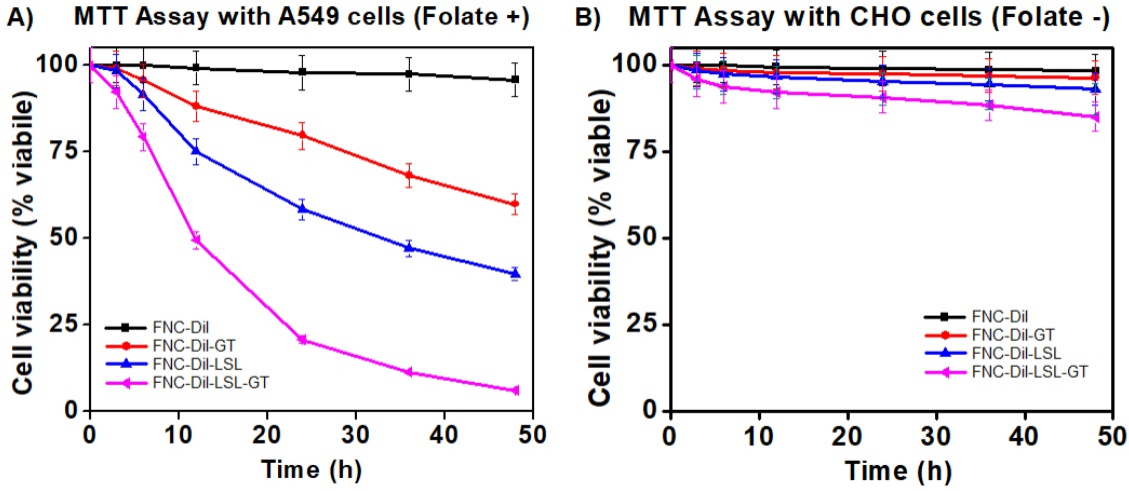
Determination of cytotoxicity of formulated folate nanoceria (FNC) using the MTT assay. **A**) More than 80% cell death occurred within 24 h when incubated with FNC loaded with both LSL and GT, whereas only 20-40% toxicity was observed when single drug was delivered. **B**) Lower toxicity was observed when drug-loaded FNC was incubated with CHO cells (FR -), suggesting that the folate receptor enabled selective uptake of drugs-carrying FNC by the cancer cell line.

To assess the therapeutic efficacy of FNC containing Dil as well as GT and/or LSL (FNC-Dil, FNC-DiI-GT, FNC-Dil-LSL and FNC-Dil-LSL-GT, 35 µL, 1.0×10^-3^ mol), a series of cytotoxicity experiments were conducted in a time dependent manner using two different cell lines, A549 (NSCLC, folate-positive) and CHO (Chinese hamster ovary, folate-negative) cells. Cells seeded in a 96-well plate (2500 cells/well), were incubated with functional NC (35 µL, 1.0×10^-3^ mol) at 37 °C. The cell viability was monitored by the MTT assay. Nanoceria by itself without drugs (**4**, FNC-DiI) showed minimal toxicity to both the cancer and normal cells. Incubation with LSL (FNC-DiI-LSL) alone resulted in about 40% NSCLC cell death at 24 h. By combining LSL and GT in FNC (FNC-DiI-LSL-GT), cell death increased 2-fold reaching 80% by 24 h and over 90% by 48 h. This result indicated for the anticancer property of LSL, which was successfully synergized by GT, where both the drugs combination (FNC-DiI-LSL-GT) showed more than 80% of cell death within 24 h of incubation, and over 90% within 48 h of incubation (**Figure 5A**). In contrast, substantial lower cell death was observed when CHO cells were incubated with drug(s) loaded FNC (**Figure 5B**) since CHO cells lack folate receptors. That is, for 48 h incubations of CHO cells with FNC-DiI-LSL-GT, cell death reached 15-20%. This demonstrates the therapeutic efficacy of LSL and importance of folate receptor that enables the targeting of cancer cell lines, thereby decreasing its cytotoxicity to normal cells. Together, the targeted delivery of FNC-DiI-LSL-GT might prove valuable in overcoming the MDR to treat undruggable NSCLC and other tumors.

### Fluorescence microscopy

**Figure 6:**
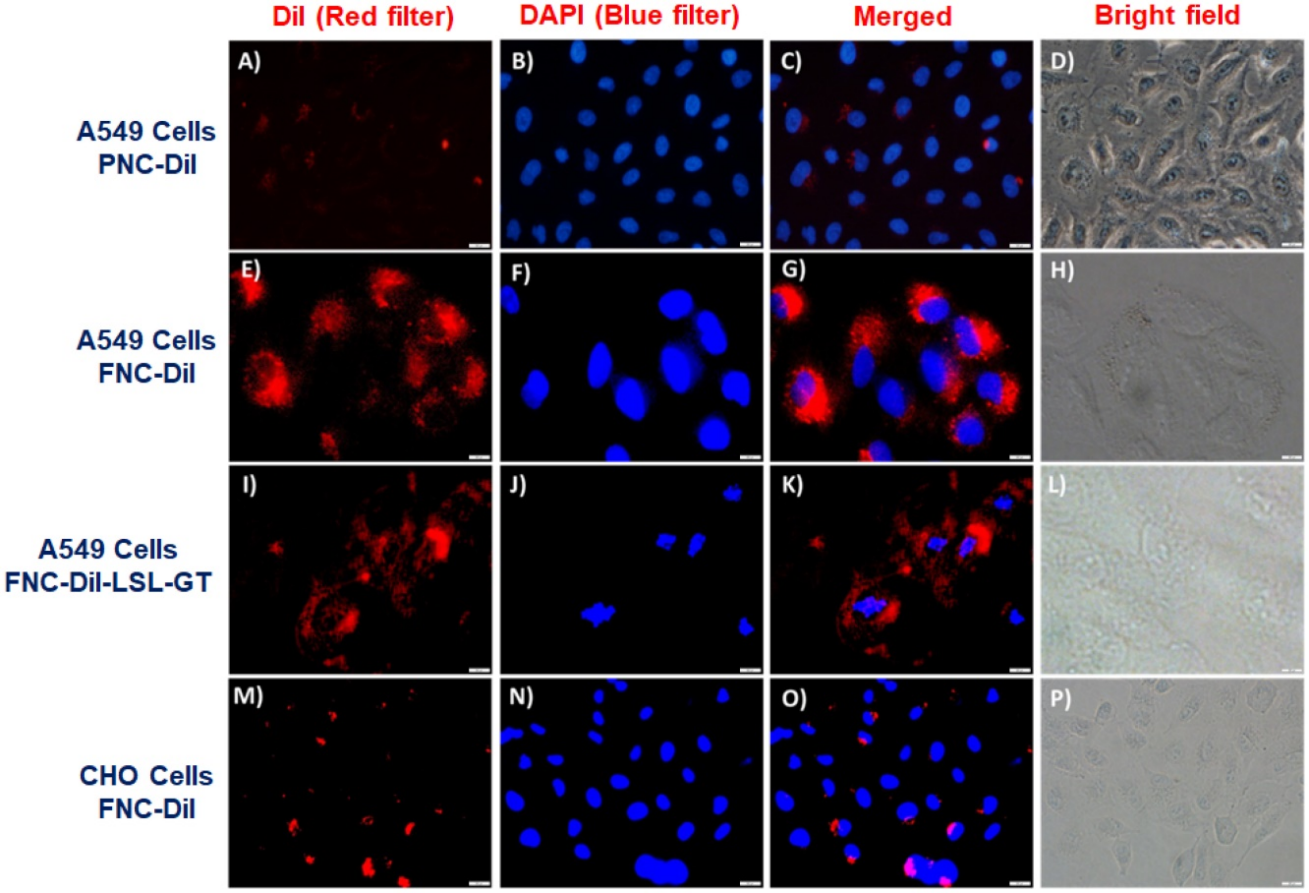
Representative fluorescence microscopic images for the *in vitro* internalizations, cellular imaging and treatment. Minimal internalization of carboxylated nanoceria was observed in A549 cells due to absence of the folate on the surface of NC (**A**-**D**, scale bar: 500 µm). Effective internalization of FNC was observed due to folate receptor-mediated endocytosis (**E**-**F,** scale bar: 200 µm). When FNC was loaded with both LSL and GT, a substantial amount of A549 cell death was observed at 24 h (**I**-**L,** scale bar: 500 µm). In contrast, minimal internalization of FNC was observed in CHO cells due to the absence of folate receptors on their surfaces (**M**-**P,** scale bar: 500 µm). Nucleus stained with DAPI dye (blue).

To interrogate receptor-mediated FNC internalizations and cargo release in living cells, we performed cellular internalization experiments and recorded a set of fluorescence microscopic images to visualize the effect of therapeutic drugs release. We hypothesized that FNC would target tumors overexpressing folate receptors. Consequently, uptake of FNC would occur to a great extent by cancer cell lines relative to normal cells decreasing cancer drug toxicity to healthy cells. When carboxylated NC encapsulated with DiI dye (**2**, PNC-DiI, 1.0×10^-3^ mol, **Figure 6A**-**6D**) was incubated with A549 cells for 24 h, low or no internalization was observed. This is due to the lack of folate ligands on the surface of PNC. However, A549 cells incubated with DiI dye encapsulated FNC (**4**, FNC-DiI) for 24 h resulted in effective internalization of FNC. This was observed due to intracellular DiI dye release that resulted in a red fluorescence cytoplasm (**Figure 6E**-**6H**). To further illustrate the folate receptor-mediated internalizations and treatment, A549 cells were incubated with FNC containing encapsulated Dil, LSL and GT (FNC-DiI-LSL-GT). After incubation for 24 h, observation of the cell morphology showed that cells had ruptured (**Figure 6I**-**6L**). These results confirm the effective receptor targeting and drug delivery to cancer cells. When CHO cells were incubated with DiI dye encapsulated FNC, minimal internalization was observed due to the lack of folate receptors on the surface of CHO cells (normal, healthy cells, **Figure 6M**-**6P**). This demonstrates that, for the selected cell lines studied herein, FNC has much higher affinity for the cancer cell line than the normal cell line. This may enable the tuning of drug doses that induce death in cancer cells while minimizing deleterious effects to normal cell lines. Taken together, these results indicate the potential of the developed FNC platform for targeted drug delivery to cancer cells.

### Cytosolic reactive oxygen species (ROS) detection assay

**Figure 7:**
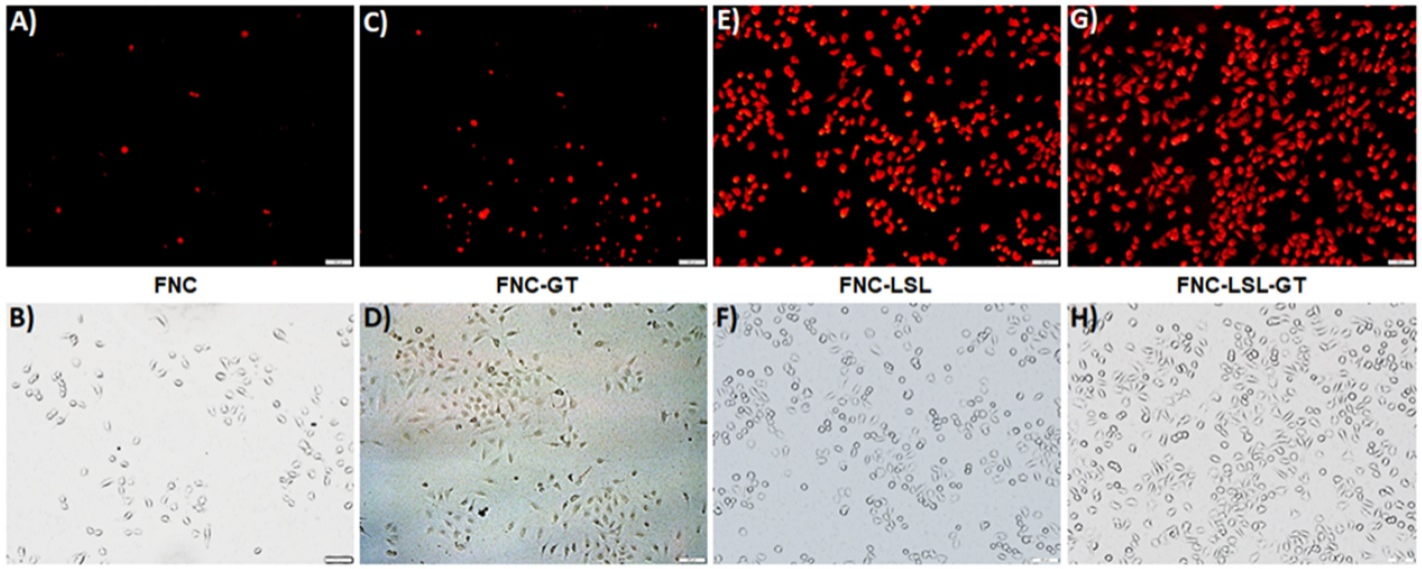
Determination of ROS generation in A549 cells when incubated with different nanoceria formulations. Increased fluorescence is due to increased ROS levels. Scale bar: 500 µm.

**Figure 8:**
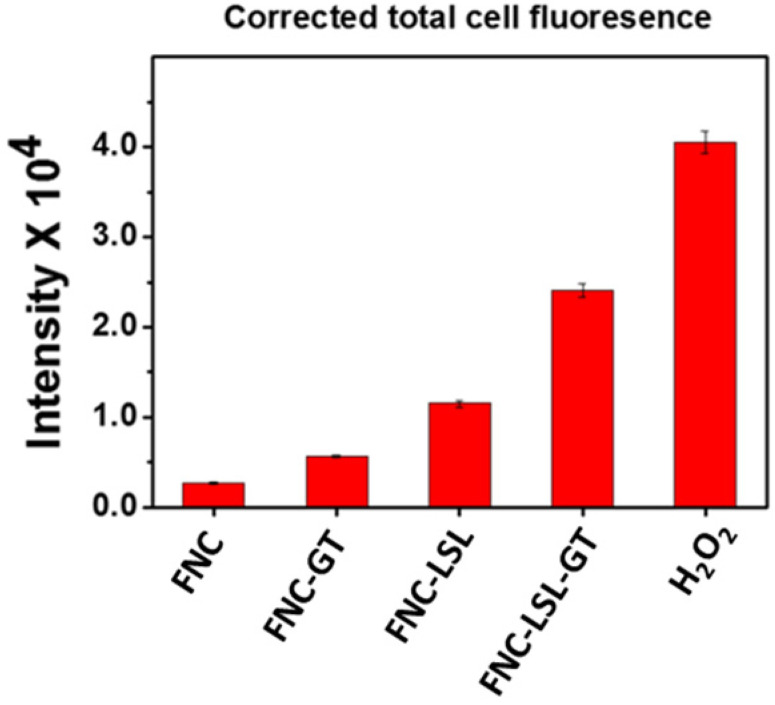
Quantification of ROS: Amount of ROS generation was quantified directly from the microscopic images using ImageJ software. Fluorescence emission results for FNC-LSL-GT relative to FNC-LSL and FNC-GT demonstrate that combining LSL-GT results in a synergistic effect on ROS generation within A549 cells.

An alternative approach to achieve better anticancer therapy is to selectively enhance reactive oxygen species (ROS) in cancer cells. The generation of ROS in the cell was determined by dihydroethidium (DHE) fluorescence, a dye which is oxidized to 2-hydroxyethidium in the presence of intracellular ROS resulting in red fluorescence. Previous studies have shown that ROS generation via HDAC inhibition as one of the mechanisms by which LSL exerts its antitumor activity 35-39. To determine, whether GT complements ROS production by LSL, A549 cells were treated with LSL and GT encapsulated within FNC (**5**, 1.0×10^-3^ mol). Briefly, A549 cells were seeded into 12-well plates (10,000 cells/well) and incubated with four different functional nanocarriers: (1) FNC without drug, (2) FNC with GT alone (FNC-GT), (3) FNC with LSL (FNC-LSL), and (4) FNC with both LSL and GT (FNC-LSL-GT). After 6 h incubations, the cells were washed and stained with the cytosolic cell permeable probe dihydroethidium (DHE) for 30 min. As shown in **Figure 7A**-**7B**, the cells treated with FNC exhibited minimal fluorescence due to limited ROS stress. FNC with encapsulated GT showed a moderate amount of fluorescence corresponding to the ROS stress induced by the drug (**Figures 7C**-**7D**). In contrast, when the A549 cells were incubated with FNC-LSL, the increased fluorescence intensity observed corresponds to an elevated ROS level (**Figures 7E**-**7F**). This may be corresponding to the higher cytotoxicity of LSL via HDAC inhibition pathway 45-49. When incubated with both LSL and GT (FNC-LSL-GT), the observed fluorescent intensity was further increased relative to FNC-LSL corresponding to the generation of even higher ROS levels (**Figure 7G**-**7H**), this further indicated that GT complemented the therapeutic efficacy of LSL by triggering ROS generation. In addition, the formation of ROS was determined by calculating the fluorescence intensities obtained from each fluorescence microscopic image using ImageJ software and the results relative H_2_O_2_ incubations are displayed in **Figure 8**. From this analysis it is evident that the additive fluorescent intensities of FNC-GT and FNC-LSL is 1.35×10^4^ whereas the fluorescent intensity of FNC-LSL-GT is 2.40×10^4^. This result demonstrates that there is a synergistic effect on ROS generation in A549 cells by combining GT and LSL.

### Apoptosis and necrosis detection assays

**Figure 9:**
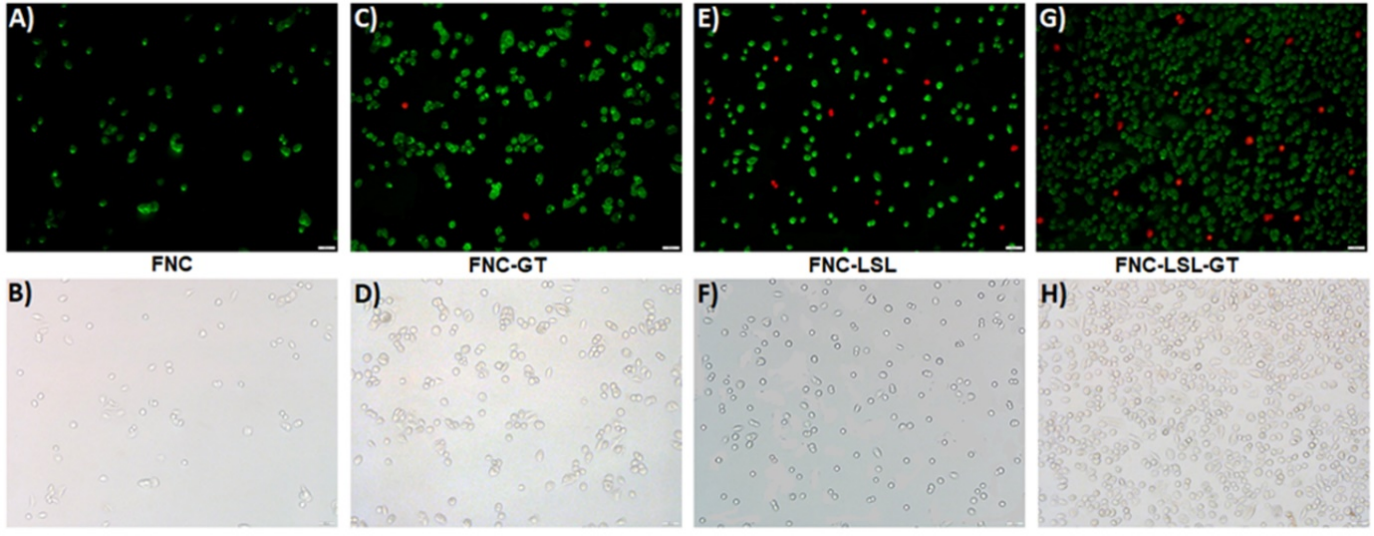
Detection of apoptotic and necrotic cell death by fluorescence microcopy using annexin V-FITC and ethidium homodimer III. **A**-**B**) Healthy cells exhibit few green fluorescence spots that correlates with the low oxidative nature of FNC. **C**-**F**) Cells incubated with FNC-LSL or FNC-GT show a substantial increase relative to FNC in the number of green fluorescence spots that correlate with induction of apoptosis. **G**-**H**) The combined encapsulation of GT and LSL with FNC results in a dramatic increase in green fluorescent spots with relatively fewer red fluorescence that correlates with a high extent of late apoptotic cells and fewer necrotic cells. Scale bar: 500 µm.

Increased ROS production often correlates with apoptosis, a form of programmed cell death. Experiments were then conducted to further elucidate the correlation between ROS generation and the induction of apoptosis. A549 cells were incubated with FNC, FNC-GT, FNC-LSL and FNC-LSL-GT (1.0×10^-3^ mol) for 24 h. Apoptotic and necrotic cells were differentiated based on their morphology and by fluorescence microscopy. Upon staining with Annexin V-FITC and ethidium homodimer III, the apoptotic and necrotic cells exhibited green and red fluorescence, respectively. This is due to that, with increased ROS levels, cells undergo various morphological and biochemical changes including translocation of phosphatidylserine (PS), leading to the induction of apoptosis. As demonstrated in **Figure 9A**-**9B,** FNC shows few apoptotic cells, which is related to the redox nature of FNC. Annexin V and ethidium homodimer stained microscopic images indicated that treatment with FNC-GT (**Figure 9C**-**9D**) and FNC-LSL (**Figure 9E**-**9F**) results in many green spots and fewer red spots that correlate with apoptotic and necrotic cells, respectively. Incubation of A549 cells with FNC-LSL-GT results in a dramatic increase in apoptotic cells (**Figure 9G**-**9H**). Hence, a synergistic effect on induced apoptosis results by combining LSL and GT, leading to extensive loss in membrane integrity, and eventually leading to cell death.

### Migration and HDAC inhibition assays

**Figure 10:**
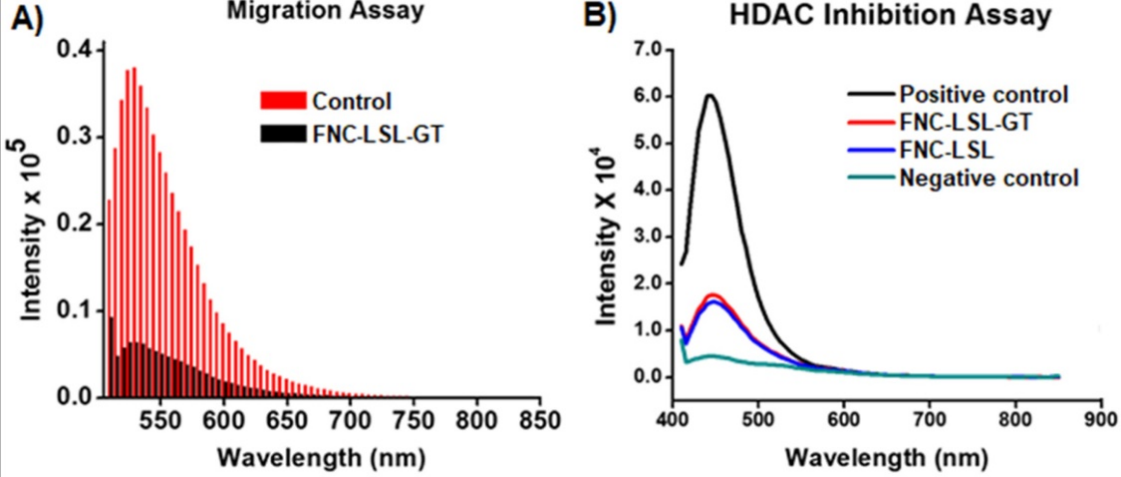
** A**) Determination of the anti-migratory effect of FNC-LSL-GT on highly metastatic lung cancer cells via the migration assay. Results indicate significant migration of cells when treated with FNC without drug incorporation (red lines). In contrast, FNC-LSL-GT (black lines) results in a dramatic decrease in the ability of the cells to migrate. **B**) Assessment of inhibition of histone deacetylase (HDAC) by lactonic sophorolipids (LSL) in comparison with positive and negative controls. As depicted, the LSL drug showed significant effect on HDAC inhibition when compared with the negative control, while GT showed minimal effect on HDAC inhibition.

Metastasis is the deadliest aspect of cancer and remains the principal cause of death despite research aimed at restricting tumor growth and migration such as what occurs during K-RAS driven NSCLC. To assess whether combining LSL with GT within FNC can inhibit tumor cell migration, we performed trans-well migration assays. In these experiments, A549 cells were serum starved and incubated with FNC (1.0×10^-3^ mol) for 24 h by seeding in the upper invasion chamber. When incubated with FNC without the incorporation of LSL or GT, cells migrate from the invasion chamber to the lower feeder tray containing 10% FBS (Control, **Figure 10A**). However, upon incubation with FNC-LSL-GT, there is a large decrease in the fluorescence intensity relative to the control that correlates with a large decrease in the migration ability of A549 cells (FNC-LSL-GT, **Figure 10A**). These results indicate that FNC-LSL-GT may play an important role in preventing the metastatic nature of NSCLC.

To assess the HDAC inhibition activity of LSL, and verify whether combining LSL and GT results in a synergistic effect, a fluorescent HDAC inhibition assay was performed. The positive control for HDAC inhibition consists of HeLa cells nuclear extract and results in the highest observed fluorescence intensity (Black line, **Figure 10B**). In contrast, the negative control Trichostatin A (TSA) results in nearly no fluorescence intensity corresponding to the absence of a HDAC enzyme (green line). FNC-LSL-GT and FNC-LSL showed significant HDAC inhibition activity when incubated with A549 cells. However, the combination of LSL and GT did not result in a synergistic effect on HDAC inhibition. Together, the observation that FNC-LSL results in HDAC inhibition indicates that this molecule follows the HDAC inhibition pathway leading to apoptosis of NSCLC cells.

## Methods

### Materials

Cerium(III) nitrate hexahydrate Ce(NO_3_)_3_.6H_2_O, polyacrylic acid (PAA), ammonium hydroxide (NH_4_OH), folic acid, N-hydroxysuccinimide (NHS), 2(N-morpholino) ethanesulfonic acid (MES) sodium salt, tetrahydrofuran, acetonitrile, sodium azide, ethanol, isopropanol and propargylamine (PA) were purchased from Acros Organics and used without further purification. Near infrared dye (DiI-D282) was obtained from Life technologies. N, N'-dimethyl formamide (DMF) and dihydroethidium (DHE) dye was purchased from Science lab and Cayman chemicals, respectively. N, N'-dimethyl sulfoxide (DMSO), esterase enzyme from porcine liver, 3-(4, 5-dimethylthiazol-2-yl) - 2, 5-diphenyltetrazolium bromide (MTT) and (1-ethyl-3-[3-(dimethylamino) propyl] carbodiimide hydrochloride) (EDC) were obtained from Sigma Aldrich and used as received. 4, 6-diamidino-2-phenylindole (DAPI-D1306) was purchased from Invitrogen. Dialysis membrane were received from spectrum laboratories. Paraformaldehyde and H_2_O_2_ were received from electron microscopy sciences. Fetal bovine serum (FBS) and 5X Annexin binding buffer were purchased from BD biosciences, whereas ganetespib, isopropyl alcohol, apoptosis and necrosis quantification kit (FITC-Annexin V, Ethidium homodimer III) were obtained from Biotium. Migration assay kit and HDAC inhibition fluorometric assay kit was obtained from Millipore and Biovision, respectively. The human lung carcinoma A549 cells (NSCLC) and Chinese hamster cells (CHO) were obtained from ATCC (U.S.A). Dulbecco's modified eagle (DMEM) medium and Kaighn's modification of Ham's F12K cell culture medium were purchased from Corning.

### Characterizations of functional nanoceria

#### Fourier transform infrared spectroscopy (FTIR)

To verify the surface functionalities of synthesized FNC, FTIR studies were performed using a Perkin-Elmer's Spectrum Two. PAA-coated NC was air dried and the powdered nanoceria was collected. FT-IR spectra of dried PAA-coated NC and PAA polymer were recorded and compared to confirm the successful coating of PAA on the synthesized NC.

#### Dynamic light scattering (DLS)

The average size distribution and surface charge of functional nanoceria were obtained using dynamic light scattering (DLS) technique. Malvern's NANO-ZS90 zetasizer was used for DLS studies. Typically, 1.0 mL of functional nanoceria was taken in a cuvette and measured for size using standard procedure set in Malvern Zetasizer. For measuring zeta potential, the sample was diluted with DI water and is carefully loaded in electrode without air bubble and the zeta potential was recorded.

#### Spectrophotometric analysis

UV-Vis and fluorescence spectroscopic studies of FNC were recorded using TECAN's infinite M200 PRO high throughput plate reader to confirm the successful conjugation of folic acid ligands on the surface of nanoceria for targeted drug delivery. Similar studies were also performed for confirming the presence of DiI dye in nanoceria for optical imaging. Procedure involves the addition of 75 µL of suspension in Thermo-Fisher 96 well plate and measuring the absorbance and fluorescence at 300-800 nm and 585 nm wavelengths, respectively.

#### *In vitro* cell culture and fluorescence microscopy

Lung carcinoma A549 cells were maintained in a 10% FBS containing DMEM medium supplemented with 1% penicillin-streptomycin antibiotic, whereas, CHO cells were propagated in 10% FBS containing Ham's F12K media containing 1% penicillin-streptomycin antibiotic. Cells were grown under 5% CO_2_ atmosphere at 37 °C in a humidified incubator. Fluorescence and bright field images of the cells were taken using Olympus IX73 fluorescence microscope.

### Synthesis of poly (acrylic acid)-coated nanoceria (1, PNC)

The synthesis of PAA-coated NC was performed following a previously published alkaline-based solvent precipitation method 15, where the cerium oxide nanoparticles were precipitated from a solution of ammonium hydroxide containing cerium salt and PAA. Briefly, solution A: cerium nitrate (0.9 g) in 2.5 mL of DI water, solution B: PAA (0.9 g) in 10 mL of DI water. Ammonium hydroxide (30 mL) was taken in a beaker, stirred at room temperature and solution A was added to it followed by the addition of solution B. A change of color from colorless to brown within 5 min of stirring was observed and finally deep yellow after 24 h, indicating the preparation of stable nanoceria. The reaction mixture is centrifuged at 3000 rpm for 30 min to obtain smaller size nanoparticles. The solution was purified by dialysis using a dialysis bag of molecular weight cut-off (MWCO) of 6-8 kDa against Di water and finally with phosphate buffer saline (PBS, pH = 7.4) to remove ammonium hydroxide and free polymer and other reagents. Final concentration of PNC was adjusted to 3.0×10^-3^ mol.

### Synthesis of propargylated nanoceria (3): Carbodiimide chemistry

Propargylated NC was synthesized by adding freshly prepared EDC (15×10^-3^ mol) solution to PNC (3.0×10^-3^ mol), followed by addition of NHS (15×10^-3^ mol) in 200 µL of MES buffer (0.1 M), pH = 6.0. The reaction mixture was incubated for 3 min at room temperature and then propargylamine (15×10^-3^ mol) dissolved in DMSO was added drop-wise and mixed. The reaction was continued for 3 h for completion at room temperature. The resultant propargylated NC was purified against PBS (pH = 7.4) using a dialysis bag (MWCO 6-8 kDa) to remove the unreacted reagents from the solution.

### Synthesis of folate conjugated NC (FNC, 4 and 5) using “click chemistry”

To the solution of propargylated NC suspension (**3**, 2.0 X 10^-3^ mol) in basic PBS solution (pH 8.0) and previously synthesized folate-azide (Fol ~ N_3_, 2.0 X 10^-2^ mol) 15, a catalytic quantity of CuI catalyst (1×10^-3^ mmol) in DMF was added and incubated on a table mixer for overnight at room temperature. The resulting folate conjugated nanoceria (FNC) was purified by dialysis to remove unreacted materials. The purified product (1.5×10^-3^) mol was stored at 4 °C for further characterizations.

### Synthesis and characterization of LSL

The synthesis of the natural glycolipid starting material LSL was performed following literature methods published by Gross's group 56,57. Briefly, fed-batch fermentation of *Candida Bombicola* ATCC 22214 inoculum aliquots (2-4×10^11^ cells/mL) was conducted maintaining the oxygen transfer rate between 50 and 80 mM O_2_/L h^-1^. The carbon sources was oleic acid (>95% purity) and glucose. 40 g of sunflower oil with high oleic acid content, 100 g of glucose, 10 g of yeast extract, and 1 g of urea in 1 L of water were used. This protocol provides product volumetric yields up to 350 g/L (productivity ~1.5 g/L h^-1^). The crude sophorolipids were extracted with ethyl acetate from the fermentation broth and dried under vacuum. The diacetylated lactonic sophorolipid, LSL, was purified by recrystallization from ethyl acetate/hexane (1:1) followed by flash chromatography using chloroform and methanol (10:1).

Yield = 83%; Purity = 97%; R_f_ [CHCl_3_/CH_3_OH 8:2] = 0.69; ^1^H NMR (500 MHz, DMSO-*d*_6_): (ppm) 1.12 (3H, d, *J* = 6.5 Hz, -C*H*_3_), 1.16-1.60 (20H, m, -C*H*_2_-), 2.00 (10H, m, -C*H*_2_CH=CHCH_2_- and 2C*H*_3_CO-), 2.30 (2H, t, *J* = 6.7 Hz, -C*H*_2_COO-), 3.05-3.15 (2H, m, H-4' and H-2”), 3.24 (1H, t, *J* = 8.4 Hz, H-2'), 3.35-3,49 (3H, m, H-3', H-5' and H-3”), 3.60 (2H, m, H-17 and H-5”), 3.98 (2H, m, H-6”), 4.06 (1H, dd, J = 11.4 and 7.0 Hz, H-6'a), 4.22 (1H, dd, J = 11.9 and 2.1 Hz, H-6'b), 4.37 (1H, d, *J* = 7.7 Hz, H-1'), 4.51 (1H, d, *J* = 7.8 Hz, H-1''), 4.71 (1H, t, *J* = 9.5 Hz, H-4''), 5.27 (1H, d, *J* = 5.1 Hz, O*H*), 5.30 (4H, m, -C*H*=C*H*- and OHs), 5.43 (1H, br s, O*H*), 5.70 (1H, br s, O*H*). ESI-MS (positive mode): m/z 711.0 (M + Na^+^).

### Encapsulation of DiI dye: solvent diffusion method

For DiI dye encapsulation, a solvent diffusion method was used. To 1.0 mL of nanoceria (PNC or FNC, 1.5×10^-3^ mol) suspension, the near infrared DiI dye (2 µL of 5 µM DiI stock in 100 µL of DMSO) was added drop-wise with continuous stirring at 1100 rpm at room temperature. The solution was incubated on table-mixer for 3 h and then the resulting solution was dialyzed against PBS (pH = 7.4) for 2 h to remove free dye from the solution. The encapsulation efficiency was measured using the following equation, EE% = [(Cargo added - Free “unencapsulated cargo”)/Cargo added] × 100, and the measurements were performed using UV-Vis spectroscopy. The purified dye-encapsulated FNC (1.0×10^-3^ mol) was stored at 4 °C for further studies.

### Co-encapsulation of lactonic sophorolipids (LSL) and ganetespib (GT)

Using a modified solvent diffusion method, the drug or combination of drugs and dye were co-encapsulated in FNC. Briefly, a solution of either LSL and DiI dye, or GT and DiI dye, or LSL/GT and DiI dye (2 µM of drug in 100 µL DMSO) was added drop-wise to 1.0 mL of vortexing FNC suspension (1.5×10^-3^ mol), followed by overnight incubation. The drug-encapsulated FNC was dialyzed against PBS (pH = 7.4) for 3 h. The purified suspension (1.0×10^-3^ mol) was stored at 4 °C for further characterizations. Presence of encapsulating cargos were confirmed by using US-Vis and fluorescence spectrophotometry.

*Encapsulation efficiency of encapsulating drugs:* To measure %EE of FNC, first concentrated solution was prepared by dissolving lyophilized FNC powder in PBS (pH 7.4). Mini dialysis cup (MWCO 6-8 kDa) was used and a smaller scale dynamic dialysis was performed to collect unencapsulated drug from outside chamber. The FNC solution (50 µL, 10×10^-3^ mol) was mixed with acidic PBS solution (100 µL, pH = 5.0) before taking in the dialysis cup. The UV/Vis absorption of unencapsulated drug was performed (**SI Figure S2**, Ganetespib λ_max_ = 254 and 288 nm, LSL λ_max_ = 256 nm) and the concentration was calculated using a standard calibration curve. The encapsulation efficiency was measured using the following equation, EE% = [(Cargo added - Free “unencapsulated cargo”)/Cargo added] × 100.

### Cargos release study

The dye release studies were carried out by using a dynamic dialysis technique at 37° C. Drug/dye loaded FNC (1.0 mL, 1.0×10^-3^ mol) was packed in a dialysis bag and suspended in 2 different environments of PBS (200 µL, pH 7.4 and 6.0). The amount of DiI dye molecules released from FNC into the outer container (Di water, 200 mL) was determined at regular time intervals by taking 1 mL aliquots from the outer container. Fluorescence emission was measured at 585 nm for DiI dye. For the drug release study in the presence of esterase enzyme, 200 μL of porcine liver esterase (1.0 mM) were added instead of PBS buffer of pH 6.0. The concentration of dye released was calculated using standard calibration curve and the cumulative release versus time was calculated using the following equation:

Cumulative release (%) = (dye released)_t_ / (dye released)_total_ × 100

where (dye released)_t_ is the amount of dye released at time t, and (dye released)_total_ is the total dye present in the dye/drug encapsulated nanoparticles.

### *In vitro* cell studies

#### Cell viability assays (MTT assay)

To determine the time-dependent cytotoxicity, two different cell lines, lung carcinoma (A549 cells) and Chinese hamster ovarian cells (CHO cells) were used. Cells were seeded in 96-well plates at a density of 2,500 cells per well and treated with various functional folate conjugated nanoceria (35 µL, 1.0×10^-3^ mol) and incubated at 37 °C for different time points. Each well was washed twice with PBS and then incubated with 50 µL of 5 mM MTT solution. After 4-6 h of incubation, the resulting formazan crystals (purple color) were dissolved in acidic isopropanol (75 µL) and the absorbance was recorded at 570 nm using TECAN's microplate reader. The assay was carried out in triplicates and the results were reported.

#### Cellular internalization study using fluorescence microscopy

Lung carcinoma cells (A549 cells) and Chinese Hamster Ovarian cells (CHO - Healthy cells) were seeded into the culture dishes and grown overnight. Once the cells are 75% confluent (10,000 cells/well), different functional drug-loaded nanoceria (PNC-DiI, FNC-DiI, FNC-DiI-LSL-GT, 50 µL, 1.0×10^-3^ mol) were treated to the cells and incubated at 37 °C for 24 h. The cells were washed thrice with 1X PBS (pH = 7.4) and were fixed with 4% formaldehyde solution for 15 min at room temperature. The cells were again washed with 1X PBS before treating with DAPI dye (5 mg/mL) for staining cell nuclei. The cells were then washed with 1X PBS and optical images were taken using fluorescence microscope for cellular internalization of dyes and killing of cells. For control experiment, CHO cells were treated with FNC-DiI-LSL-GT (50 µL, 1.0×10^-3^ mol) and the images were captured using a fluorescence microscope.

#### Cytosolic ROS detection assay

To know the mechanism of cell death, reactive oxygen species (ROS) assay was performed. This assay determines the process of induction of stress to the cells by the generation of ROS. A549 cells were seeded in 12-well plates at a density of 10,000 cells per well and treated with FNC encapsulated with different drugs (50 µL, 1.0×10^-3^ mol) and incubated at 37 °C. After 6 h of incubation, the cells were washed thrice with 1X PBS (pH = 7.4) and stained with 20 µL of DHE (32 µM) fluorescent probe for about 30 min at room temperature followed by washing the cells twice with 1X PBS. Subsequently, the cells were fixed with 1 mL of paraformaldehyde solution. After fixation, the cells were washed with 1X PBS, stored with 2 mL PBS in each well and images were taken using fluorescence microscope.

#### Quantification of ROS fluorescent images

Using ROS fluorescent images, the amount of ROS generated (equivalent to red fluorescence) was quantified using commercial ImageJ software*.* Particular cell from each treated well was selected to get a stack of values for the area, integrated density, and mean fluorescence of background readings. The corrected total cell fluorescence (CTCF) for each well was calculated using the formula: CTCF = integrated density - (area of selected cell X mean fluorescence of background readings).

#### Apoptosis and necrosis detection assay

For the assessment of apoptosis and necrosis, Annexin V-FITC and Ethidium homodimer III kit was used. Briefly, A549 cells were seeded in 12-well plates at a density of 10,000 cells per well. Next, cells were treated with different preparations (FNC, FNC-LSL, FNC-GT and FNC-LSL-GT, 50 µL, 1.0×10^-3^ mol). After 6 h of incubation, the cells were washed twice with 1X PBS (pH = 7.4) and stained with two different dyes, 5 µL of FITC-annexin buffer and 5 µL of ethidium homodimer III and incubated for 15 min in the dark. Later the cells were washed twice with 1X binding buffer and fixed with 4% formaldehyde solution and the cells were covered with 1X binding buffer. Multiple fluorescence images were taken using two filters: green fluorescence representing apoptosis, red fluorescence indicating necrosis.

#### Migration assay

Experimental procedure involves the incubation of serum starved A549 cells for 24 h. Next, the cells were harvested and treated with FNC and drugs-loaded FNC (50 µL, 1.0×10^-3^ mol). Subsequently, the cells were seeded in invasion chamber of the migratory assay kit coated with collagen layer. The feeder tray contained 10% FBS media and the complete set up was incubated at 37 °C for 24 h to allow the migration of cells. Later, the migratory cells were dislodged completely from the invasion chamber and placed on new feeder tray containing cell detachment buffer and incubated for 30 min. Finally, diluted solution of CyQuant GR dye and cell lysis buffer was added to stain migratory cells and the fluorescence intensity was measured at an emission wavelength of 520 nm, using a microplate reader.

#### HDAC inhibition assay

HDAC fluorometric assay kit was used to determine the HDAC inhibition ability of lactonic sophorolipids (LSL). Assay was performed according to manufacturer's protocol. HeLa nuclear extract and Trichostatin A were used as positive and negative controls respectively. Briefly, A549 cells were seeded in a 96-well microtiter plates and after 80% confluence (2,500 cells/well), the cells were treated with functional nanoceria (50 µL, 1.0×10^-3^ mol) and incubated at 37 °C for 24 h. Positive and negative controls were added along with HDAC assay buffer and the reaction was initiated by adding HDAC fluorometric substrate, and incubated for 30 min. Later, the HDAC lysine developer was added to stop the reaction and was incubated for additional 15 min. The fluorescence intensity was then measured at 460 nm using a microplate reader.

## Conclusions

In summary, we successfully demonstrated the potential anticancer property of LSL using folate functionalized nanoceria (FNC) for the targeted treatment of K-RAS driven NSCLC. Characterization studies on FNC showed its enhanced stability in PBS at physiological conditions. The functionalization of surface carboxylic acids with folate allowed targeting and, consequently, enhanced uptake by the non-small cell lung cancer (NSCLC) cell line A549, an epithelial carcinoma that is a KRAS mutant. Formulated nanoceria showed higher encapsulation efficiency of drugs. Results in this work demonstrate that by combining LSL with GT inside the FNC nanocarrier, these drugs synergistically enhanced hsp90 inhibition, as confirmed by several cell-based assays. Fluorescence microscopy images confirmed the successful receptor-mediated cellular targeting and cell internalization of A549 cells using folate functionalized NC. FNC-LSL-GT caused an 80% decrease in A549 cell viability during a 24 h incubation. Furthermore, the mechanisms of action of FNC-LSL-GT involved Hsp90 and HDAC inhibitions that resulted in the enhanced intracellular ROS that facilitated the apoptotic pathway. Furthermore, FNC-LSL-GT demonstrated metastasis suppressing activity based on a mobility assay. We conclude, based on the *in vitro* assays conducted herein to assess the anti-cancer activities of LSL and FNC-LSL-GT on the A549 cell line, that the reported results are encouraging and warrant further investigation of the potential of this therapeutic system for the treatment of NSCLC and related cancer diseases.

## Supplementary Material

Supplementary figures.
