# Supplementary material for: Therapeutic Efficacy of Lactonic Sophorolipids: Nanoceria-Assisted Combination Therapy of NSCLC using HDAC and Hsp90 Inhibitors
Source: Nanotheranostics. 2021 Apr 16;5(4):391–404. doi: 10.7150/ntno.57675 (PMC8077971; doi:10.7150/ntno.57675)
Supplement: Supplementary file 1 — Supplementary figures. [file ntnov05p0391s1.pdf]

Electronic Supporting Information

Therapeutic Efficacy of Lactonic Sophorolipids: Nanoceria-Assisted Combination Therapy of NSCLC using HDAC and Hsp90 Inhibitors

Shugufthа Naz[1], Tuhina Banerjee[1], Filbert Totsingan[2], Kalee Woody[1], Richard A. Gross[2], Santimukul Santra[1,*]

1. Department of Chemistry, Pittsburg State University, Pittsburg, Kansas 66762, United States.

2. Department of Chemistry and Chemical Biology, Rensselaer Polytechnic Institute, Troy, New York 12180, United States.

* Corresponding author: Santimukul Santra, Ph.D., Associate Professor. Department of Chemistry, Pittsburg State University, Pittsburg, Kansas 66762, United States. Phone: 620-235-4861, Fax: 620-235-4003. Email: ssantra@pittstate.edu

1. Characterization of functional nanoceria by FT-IR spectroscopy.

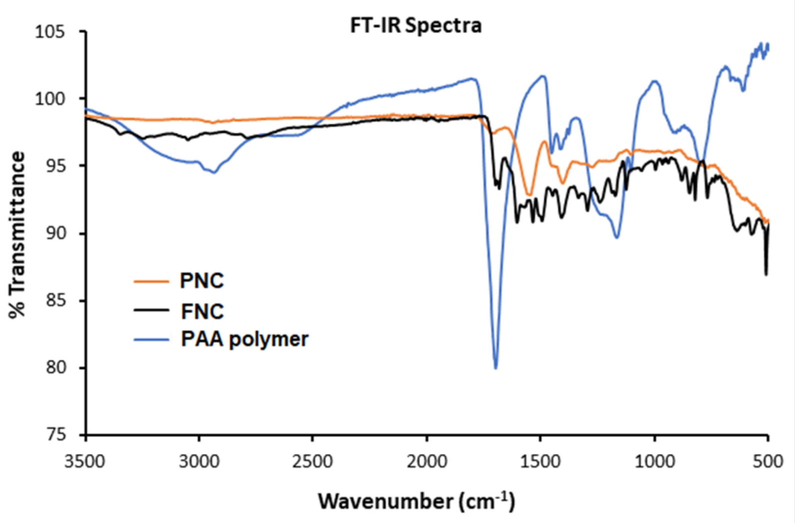

Figure S1. FT-IR spectra of polyacrylic acid polymer (PAA), PAA-coated nanoceria (PNC) and folic acid conjugated nanoceria (FNC).

2. **Drug encapsulations by UV-Vis spectroscopy.**

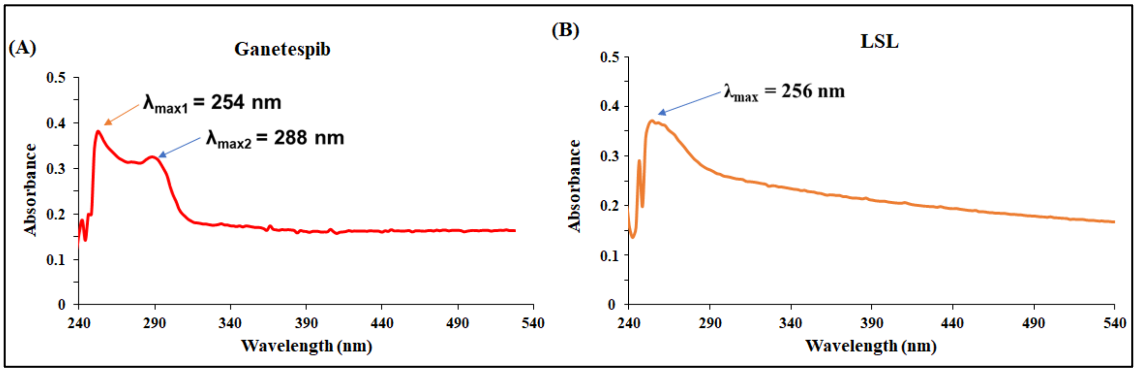

Figure S2. UV-Vis spectra (normalized) of encapsulated drugs, A) Ganetespib and B) LSL, after digestion of FNC (1.0 X 10^{-3} mol) using PBS buffer at pH 5.0.

ToC Figure:

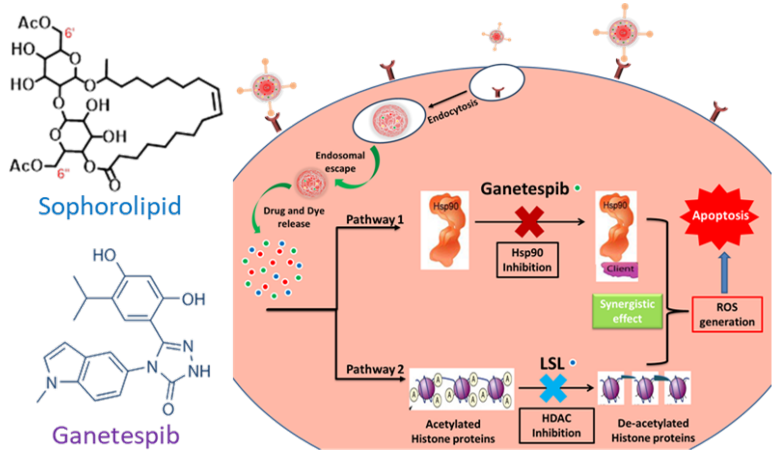

Sophorolipid
Ganetespib
Endocytosis
Endosomal escape
Drug and Dye release
Pathway 1
Pathway 2
Ganetespib
Hsp90
Hsp90 Inhibition
Client
Apoptosis
Synergistic effect
ROS generation
LSL
Acetylated Histone proteins
HDAC Inhibition
De-acetylated Histone proteins
